# A Gamified mHealth App to Promote Physical Activity and Reduce Sedentary Behavior in Autistic Adults: Protocol for a Remotely Delivered Pilot Intervention Study

**DOI:** 10.2196/71631

**Published:** 2025-07-22

**Authors:** Daehyoung Lee, Lia McNulty, Swetha Kathiravan, Matthew Louis Mauriello, Vijay Vasudevan, Anjana Bhat, Iva Obrusnikova, Richard Suminski

**Affiliations:** 1 Department of Health Behavior and Nutrition Sciences College of Health Sciences University of Delaware Newark, DE United States; 2 Department of Computer and Information Sciences College of Engineering University of Delaware Newark, DE United States; 3 Data Science and Evaluation Autism Speaks Washington, DC United States; 4 Department of Physical Therapy College of Health Sciences University of Delaware Newark, DE United States

**Keywords:** gamification, autism, physical activity, mobile health, participatory research

## Abstract

**Background:**

Research indicates that many autistic adults are insufficiently active and overly sedentary. There is limited evidence on effective strategies to increase physical activity (PA) and reduce sedentary behavior (SB) in this population. Gamified mobile health (mHealth) interventions show promise for addressing these challenges by leveraging autistic individuals’ strengths in visuospatial learning and their affinity for digital gaming. Despite this potential, it remains unclear how well these interventions translate to real-world settings. This gap is compounded by the lack of community-based participatory approaches in the development of mHealth intervention for autistic adults.

**Objective:**

This study aims to (1) formulate gamification and behavior change strategies for the PuzzleWalk v2 app using a community-based participatory approach and (2) evaluate its feasibility and acceptability for increasing PA and reducing SB among autistic adults, including those with mild intellectual disability, in real-world settings.

**Methods:**

This study, consisting of 2 sequential phases, will be conducted entirely remotely: (1) online community-based design workshops to refine the PuzzleWalk gamified mHealth system with input from key autism stakeholders, including autistic adults and caregivers, and (2) an 8-week field deployment to assess real-world usability and engagement. In Phase I (completed), weekly workshops and usability testing focused on understanding autistic adults’ technology preferences, evaluating PuzzleWalk v1 and v2 prototypes, and incorporating stakeholder feedback into iterative app development (n=9). Phase II (in progress) will involve a single-arm clinical trial where approximately 70 participants will use the app alongside research-grade activity-tracking accelerometers to measure PA and SB. Outcome measures, including sedentary time, step counts, PA intensities, and app engagement (eg, time spent using the app), will be collected across 4 specific time points (ie, baseline and weeks 3, 5, and 8). Repeated measures ANCOVA will be performed to examine changes in participants’ objective levels of PA and sedentary time before, during, and after the intervention.

**Results:**

Phase I of the study, involving community-based participatory design workshops and usability testing, was completed in November 2024. Key autism stakeholders recognized the gamified PuzzleWalk app as a viable tool for enhancing motivation toward PA and SB changes among autistic adults. Data collection for Phase II, the field deployment, is currently underway and is expected to end in August 2025. As of July 2025, we had enrolled 69 participants in the study. The findings of these studies will be shared in a subsequent peer-reviewed publication.

**Conclusions:**

Results of the ongoing field deployment study (Phase II) will further clarify the app’s effectiveness and real-world applicability.

**Trial Registration:**

ClinicalTrials.gov NCT06566131; https://clinicaltrials.gov/study/NCT06566131

**International Registered Report Identifier (IRRID):**

DERR1-10.2196/71631

## Introduction

### Background

Increased awareness and advancements in diagnostic methods have made autism spectrum disorder (ASD) one of the most rapidly growing developmental disabilities in the United States [1,2]. However, poor health outcomes among autistic adults have emerged as a significant public health concern. Approximately 5.4 million American adults were diagnosed with this lifelong neurodevelopmental condition, with or without co-occurring intellectual disability [3]. Autistic adults are at higher odds of lifestyle-related chronic health conditions, including obesity (1.4-fold), dyslipidemia (2.1-fold), and diabetes (2.2-fold) [4,5].

The *Healthy People 2030* initiative highlights the importance of regular physical activity (PA) participation for maintaining overall health among individuals with and without disabilities. However, more than one-quarter of American adults, including autistic adults, participate in no leisure-time PA [6]. Research findings indicate that individuals with ASD often exhibit lower PA levels and increased sedentary behavior (SB) with age, potentially due to diminished intrinsic motivation to move and heightened sensory sensitivities in public environments [7,8]. Moreover, evidence [9] categorizes SB as a distinct health risk factor, as prolonged sitting contributes to adverse health outcomes even among those meeting PA guidelines (ie, accumulating ≥150 minutes a week of moderate-to-vigorous PA [MVPA] or ≥75 minutes a week of vigorous aerobic PA) [10]. Physical inactivity and excessive sitting may contribute to the high prevalence of chronic health conditions in autistic adults [4]. Despite these concerns, strategies to enhance preventive health behaviors in autistic adults remain under-researched. Studies investigating PA and SB in autistic adults frequently show methodological limitations, such as small sample sizes and the underrepresentation of individuals with co-occurring intellectual disability [11].

Gamified mobile apps hold promise for effectively promoting positive changes in PA and SB in autistic adults by leveraging their unique strengths in visuospatial learning and digital gaming [12-15]. It is well documented that technology particularly appeals to autistic adults because it provides a structured, consistent experience with lower social demands than face-to-face communication [13,16]. Gamified behavioral interventions delivered via smartphone apps have rapidly expanded the potential for improving health behaviors in a way that is engaging and cost-effective. The gamification approach can enhance a user’s perception of autonomy, fostering intrinsic motivation and contributing to enjoyable experiences, which collectively sustain their engagement [17]. However, most evidence on the efficacy of gamification approaches in promoting free-living PA comes from studies involving typically developing young people, with a lack of rigorously designed empirical studies involving autistic populations [18]. Financial incentive–based apps have also emerged as a viable strategy for increasing PA and reducing sedentary time, particularly for individuals with low motivation for voluntary behavior change [19]. Although preliminary evidence suggests that such apps can improve PA and reduce SB in both autistic and nonautistic groups, questions remain about the long-term sustainability of these interventions. Early versions of gamified mobile health (mHealth) apps, such as PuzzleWalk v1, laid a significant foundation as innovative, strength-based interventions for autistic adults, but they did not yield long-term improvements in PA and SB changes in a small sample of autistic adults [20]. Existing mHealth interventions aimed at promoting PA often encounter pragmatic barriers, including difficulty of use, limited system integration, poor scalability, and lack of personalization, which can lead to reduced user engagement and adherence [21-23]. These challenges may be more pronounced for autistic adults who often face additional barriers related to sensory sensitivities and the need for structured routines and consistent feedback [4,7,8]. Furthermore, few efforts have been made to integrate community input during the design and development of mHealth apps [18]. This gap directly informed this study, which centers on the co-design of PuzzleWalk v2 to enhance both real-world usability and sustained engagement for autistic adults.

While most studies on PA have disproportionally focused on young adults (eg, 18-30 years old) without co-occurring intellectual disability, a substantial proportion of young autistic adults remains insufficiently active [11,24]. Current evidence on interventions designed to promote PA and reduce SB in autistic adults remains limited and is frequently characterized by poor methodological rigor in the development and evaluation of intervention strategies [11]. Gamified mHealth interventions represent a promising approach for facilitating positive PA and SB changes by leveraging the distinct strengths and preferences of autistic individuals. Addressing adoption challenges and evaluating the real-world applicability of gamified mHealth interventions are essential for improving preventive health behaviors within this community.

### Study Aims

This paper presents a study protocol for the remote co-design and field-testing of a novel mHealth system, guided by gamification and behavior change theories. The overarching objective of this study is to develop an effective and sustainable mHealth intervention that promotes regular PA participation and reduces SB in autistic adults using a community-based participatory research approach. The study’s specific aims are to (1) develop practical gamification strategies that promote PA and SB changes in autistic adults and (2) evaluate the feasibility and acceptability of the PuzzleWalk v2 mobile app in increasing free-living PA and reducing SB among autistic adults. Developed by the corresponding author’s research team, PuzzleWalk v1 demonstrated the potential to induce short-term behavioral changes in a previous feasibility deployment study [20]. Building on these preliminary findings, this study will refine evidence-based design principles to promote sustained improvements in PA and SB in autistic adults, including those with and without mild intellectual disability.

## Methods

### Study Design

This study comprises 2 sequential phases: (1) online community-based participatory design workshops with autism community stakeholders (Phase I; completed) and (2) an 8-week field deployment of the PuzzleWalk v2 mHealth system in real-world settings (Phase II; in progress).

#### Phase I: Community-Based Participatory Design Workshop

The first phase involved weekly online design workshops with key community stakeholders, including 7 autistic adults, 1 parent or caregiver, and 1 speech therapist with over 15 years of experience working with individuals with ASD. These remote workshop sessions utilized individual interviews and online surveys to evaluate preferences and technology usage patterns, identify applicable gamification and behavior change strategies to enhance engagement and adherence, and review the app’s previous (v1) and current (v2) functional prototypes. Specifically, the workshop sessions focused on (1) exploring technology-related factors that influence PA participation and prevalent SBs in autistic adults, (2) discussing the current features of PuzzleWalk v1 (eg, strengths, weaknesses, and opportunities for improvement), and (3) identifying best-practice intervention strategies to address challenges associated with autism symptoms and enhance user adherence during field deployment (Table 1). Meeting formats were adapted to accommodate the diverse needs and preferences of stakeholders based on the Academic Autistic Spectrum Partnership in Research and Education (AASPIRE) guidelines [25]. Data from interviews and surveys were analyzed using frequency and narrative data analysis methods. The core findings were shared with the software development team and iteratively incorporated into the PuzzleWalk v2 system. The multiformat co-design workshop guide provides the core questions and discussion prompts used throughout the participatory design phase (see Multimedia Appendix 1). Finally, pilot usability testing was executed with various iPhone models to ensure the app’s technical compatibility and functionality before field deployment.

**Table 1:** Focused activities in community-based participatory design workshops.

Timeline	Activities	Format
Week 1	Screening interviews and general inquiry on demographics, patterns of PA^a^ and SB^b^, and overall health among key stakeholders with ASD^c^	Phone call or virtual meeting
Week 2	Exploration of preferences and patterns of general technology use and its influence on PA and SB among autistic adults	Survey or email
Weeks 3-4	Identification of factors influencing PA participation and perceived health benefits of PA	Survey or email
Weeks 5-6	Evaluation of the core features (eg, step-to-game conversion) and identification of areas for improvement in PuzzleWalk v1	Virtual meeting
3-month period	Integration of key findings into PuzzleWalk v2	Not applicable
Weeks 7-8	Autism-friendly intervention design and pilot compatibility and functionality testing of PuzzleWalk v2 in real-world settings^d^	Survey or email

^a^PA: physical activity.

^b^SB: sedentary behavior.

^c^ASD: autism spectrum disorder.

^d^5 stakeholders who own an iPhone participated in this activity.

#### Phase II: Field Deployment of PuzzleWalk v2 mHealth System

Autistic adults who consent to participate in this single-arm clinical trial (NCT06566131) will receive study materials via the United States Postal Service, including a research-grade accelerometer/monitor (ie, ActiGraph CentrePoint Insight wristwatch), a cable charger, and study instruction sheets. Participants will also receive visual guidelines for wearing the monitor and returning study materials. The monitor wear guide will instruct the participants to wear the monitor comfortably on their nondominant wrist during waking hours, removing it only for water-related activities such as showering or swimming. Each night before bed, participants will record their monitor wear time in their tracking log and remove the watch during sleep. Reminders will be sent to participants every 3 weeks to 4 weeks to ensure uninterrupted use per the manufacturer’s recommendation for the monitor’s charging. The return guide will instruct participants to return the study materials by placing the monitor, charger, and tracking logs in a provided Ziplock bag, sealing them in a prepaid Priority Mailbox, and mailing them back to the research team via United States Postal Service (see Multimedia Appendix 2). Upon receipt, the research team will confirm the return by text message or email.

**Figure 1:**
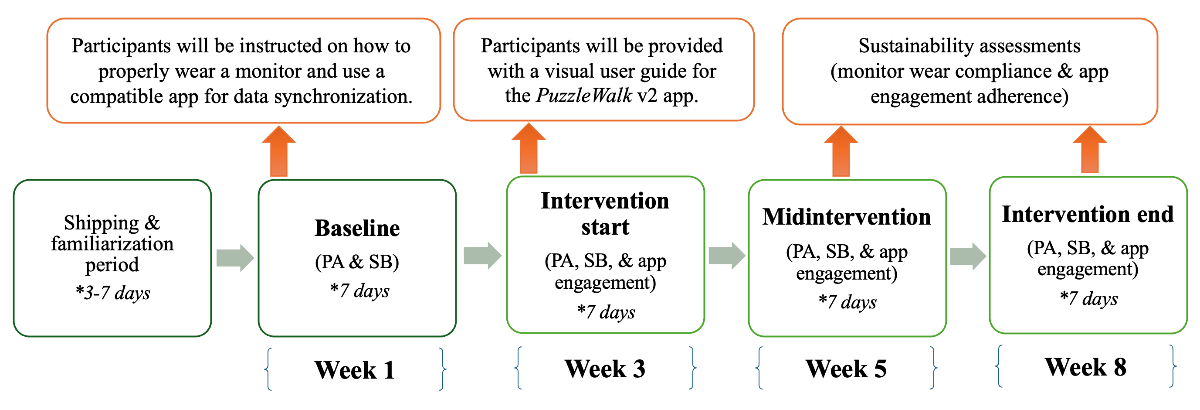
Data collection flowchart in Phase II. PA: physical activity; SB: sedentary behavior.

Before baseline data collection, we will provide a ramp-up familiarization period to help participants understand the study protocol and requirements, and to ensure they are comfortable wearing the activity-tracking accelerometer on their nondominant wrist. Throughout the deployment period, the research team will use SimpleTexting to communicate regularly with study participants and to promptly respond to any questions they have. SimpleTexting is an SMS text messaging service provider that allows the research team to send mass text messages and engage in real-time conversations with participants. Field data collection will begin once the participant completes a demographic survey and confirms receipt of the study package. This includes gathering data on PA and sedentary time using ActiGraph CentrePoint Insight watches and app engagement (eg, time spent using the app and number of session logins) using Voyager, an open-source Laravel administration package (The Control Group). The Voyager database management system will enable the research team to monitor user engagement and manage user profiles in near real time. Specifically, this administration system will be used to identify participants’ mobile devices and update their unique usernames on the leaderboard of PuzzleWalk v2. Starting week 3, participants will begin using PuzzleWalk v2, following a visual, step-by-step user guide, and continue using the app until the end of week 8 (total 6-week intervention). Outcome measures will be collected during 4 time periods: week 1 (baseline), week 3 (intervention start), week 5 (midintervention), and week 8 (intervention end). Periodic reminders and reinforcement messages, incorporating evidence-based behavior change techniques, will support their continued engagement with the app and PA participation (Figure 1).

### Sample and Recruitment Strategies

This pilot study acknowledges the potential for selection bias because individuals who volunteer for this PA-related study may already have a relatively high level of motivation to change their behavior. According to a priori power analysis using G*Power 3.1 [26], considering an α level of .05, an estimated effect size (f) of 0.2, an anticipated correlation of 0.4 among repeated measures, and 4 measurement levels of within-subjects factors, we project a total sample of 55 autistic adults to achieve a power of 0.9. To be eligible, prospective participants must (1) be adults aged 18 years to 55 years, (2) have received a formal diagnosis of ASD from a qualified medical professional, and (3) own and regularly use a compatible iPhone (iOS 14+ operating system). Android version development is underway, and its users will be invited to participate in future clinical trials. Individuals with moderate-to-profound intellectual disability or orthopedic conditions that significantly interfere with independent information processing or ambulation (eg, using a wheelchair or crutches) will be excluded from the study. These individuals may encounter challenges in engaging with the cognitive and physical aspects of the intervention, such as understanding verbal or written instructions, independently using the app, or meeting study requirements (eg, returning study materials to the research team). Therefore, only individuals who live semi-independently or independently, have autonomy for daily living, and are capable of making their own research-related decisions will be given an informed consent form to sign and subsequently invited to participate in the study. Future research shall explore adaptation strategies to make the mHealth interventions more accessible to a broader autistic population, including those with co-occurring moderate-to-profound intellectual disability.

All participants will provide written or digital consent before participating in study activities. The research team established a multisource recruitment model that leverages national and regional autism advocacy organizations (eg, Autism Speaks, Autism Delaware), Simons Foundation Powering Autism Research for Knowledge (SPARK) research match services, ResearchMatch.org, and online autism support groups on social media (eg, Reddit and Facebook). The study flyer will be posted on the Autism Speaks research search portal (*approval pending*), ResearchMatch.org (*approved*), and relevant group pages on social media channels (eg, Autism Delaware Facebook page; *approved*). Individuals interested in participating in the study can contact the research team directly and undergo a screening interview to verify their eligibility. SPARK’s research match service (*approved*) will identify potential participants based on study criteria and send them study invitations. Our research team will be provided with the diagnostic and contact information of those who consent. Individuals recruited through other channels will undergo a screening interview to confirm their eligibility. The interview will explore details about when and how the ASD diagnosis was made, as well as common autism symptoms. Based on the results from preliminary studies [20,27,28], we expect to recruit all eligible participants within the first 3 months of the study using this multisource recruitment model.

### Ethical Considerations

The study was reviewed and approved by the University of Delaware Institutional Review Board (IRB #2098388), with all procedures adhering to institutional and national ethical standards for research involving human participants. In Phase I, which has been completed, all participants provided written or digital informed consent before engaging in remote community-based design workshops and usability testing. Only individuals with sufficient cognitive and communicative capacity to provide autonomous consent were enrolled. Data collected during this phase were de-identified prior to analysis, and any identifiable information, such as email addresses, was promptly destroyed following data collection. Participant privacy was protected using encrypted communication protocols and secure data storage systems accessible only to authorized personnel, such as the principal investigator and their research assistants.

Phase II, the ongoing field deployment, involves similar ethical safeguards. Participants provided informed consent prior to receiving study materials, including accelerometers and study instructions. They were fully briefed on the study protocol and expectations. Participants are being compensated with a US $100 Amazon e-gift card upon verified completion of study requirements, including consistent accelerometer wear and tracking log submissions over the 8-week data collection period. Additional incentives in the form of e-gift cards are offered to top performers on the app’s gamified leaderboard to encourage engagement. All data collected during this phase continue to be anonymized and securely managed through access-controlled systems. No identifiable participant images or information will be included in future manuscripts or supplementary materials.

### PuzzleWalk mHealth System

**Figure 2:**
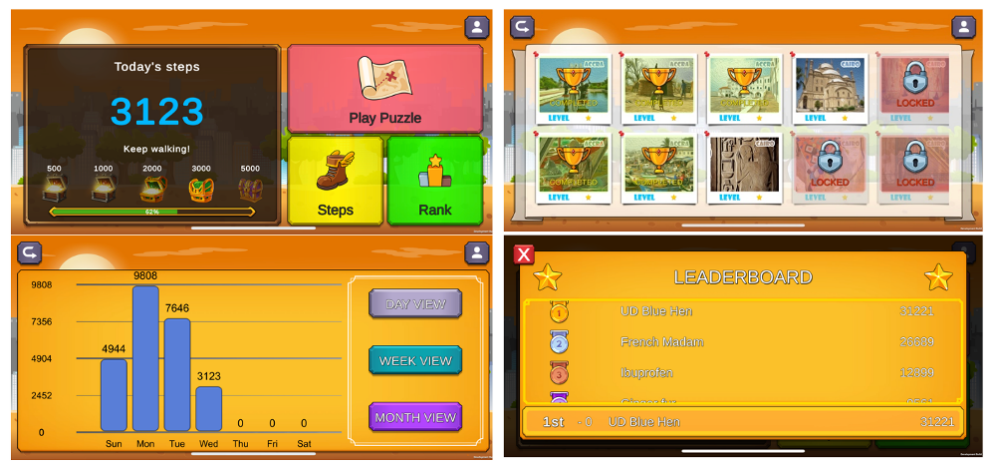
Functional interfaces of PuzzleWalk v2.

PuzzleWalk is a gamified, behavior change theory–guided mobile app designed to promote regular PA participation among autistic adults by leveraging practical behavior change techniques and principles of self-determination theory [12,20,28]. Key innovative features include a step-to-game conversion algorithm, which translates users’ daily step counts into time for solving image-based puzzle games, highlighting iconic cityscapes from around the world. This user-centered design builds on the strengths of autistic individuals in visual learning and fosters intrinsic motivation through autonomy, relatedness, and competence. To achieve these core elements of self-determination theory, PuzzleWalk incorporates self-monitoring of step counts to support autonomous app engagement, visual puzzle games, contingent rewards to enhance competence, and a competition-based leaderboard and shared goals to foster relatedness among autistic users. PuzzleWalk v2 builds on the original version through iterative design enhancements guided by feedback from key stakeholders during the participatory workshop phase. A major advancement in PuzzleWalk v2 is the inclusion of improved accessibility features, such as step-by-step visual guides, simplified interface navigation, and animated icons and buttons to enhance user-friendliness. Another key improvement from v1 to v2 is the introduction of a gamified goal-tracking feature that uses a quest-based design, allowing users to unlock puzzle rewards by reaching step count milestones (Figure 2). The leaderboard scores are determined by combining accumulated step counts and puzzle points. Top scorers on the leaderboard are eligible to receive tangible incentives (eg, Amazon e-gift cards) at the end of each month. Importantly, the PuzzleWalk system automatically resets leaderboard scores at the end of each month to encourage consistent PA engagement among users. Detailed descriptions of the PuzzleWalk v1 system can be found in previous studies [20,28].

### PA and SB Assessment

PA, including daily step counts, light PA, and MVPA, as well as sedentary time, will be measured using research-grade CentrePoint Insight Watches (ActiGraph). Triaxial accelerometers by ActiGraph have been widely used to provide objective assessments of PA and sedentary time, demonstrating moderate-to-high reliability across diverse clinical populations [29]. All participants will wear an accelerometer on their nondominant wrist during waking hours, except during water activities. They will be instructed to continuously wear an accelerometer for 7 consecutive days, capturing behavioral data from at least 3 weekdays and 1 weekend day across the 4 data collection intervals (ie, weeks 1, 3, 5, and 8). Compliance will be supported through regular check-ins and reminders.

Accelerometers will be programmed to collect data in 60-second epochs. Levels of activity intensity and sedentary time will be classified using activity counts per minute (CPM) cut point values: sedentary (<2860 CPM), light PA (2860-3940 CPM), and MVPA (≥3941 CPM) [30]. These cut point thresholds, established by Montoye et al [30], underwent cross-validation in both structured laboratory and free-living conditions with adults. A valid day will be defined as a minimum of 10 hours of accelerometer wear time a day. To be included in the data analysis, participants must have at least 3 valid days of accelerometer wear during each data collection period [31]. Despite the absence of a definitive consensus on the required number of valid days, previous research commonly accepts 3 days to 4 days as adequate for reliable estimation of one’s general patterns of PA engagement [32,33]. Nonwear time will be defined as 90 minutes of consecutive zero activity counts, with a 2-minute spike tolerance [34]. The accelerometers will be programmed to record PA and sedentary time at a default sampling frequency of 32 Hz. The CentrePoint cloud portal will streamline management and initial analysis of accelerometry data through a manufacturer-provided mobile app (ie, CentrePoint Connect app). Given the project’s timeline, participants will be required to install the app on their devices to remotely synchronize accelerometry data and prevent data loss before the device storage capacity (ie, 30 days or 512 MB) is reached. Processed raw accelerometry data will be securely managed and stored in the customizable CentrePoint cloud, and the research team will have a real-time overview of participant compliance (eg, at least 10 hours of accelerometer wear per day), data outcomes, and overall study progress.

**App Engagement Assessment**

**Figure 3:**
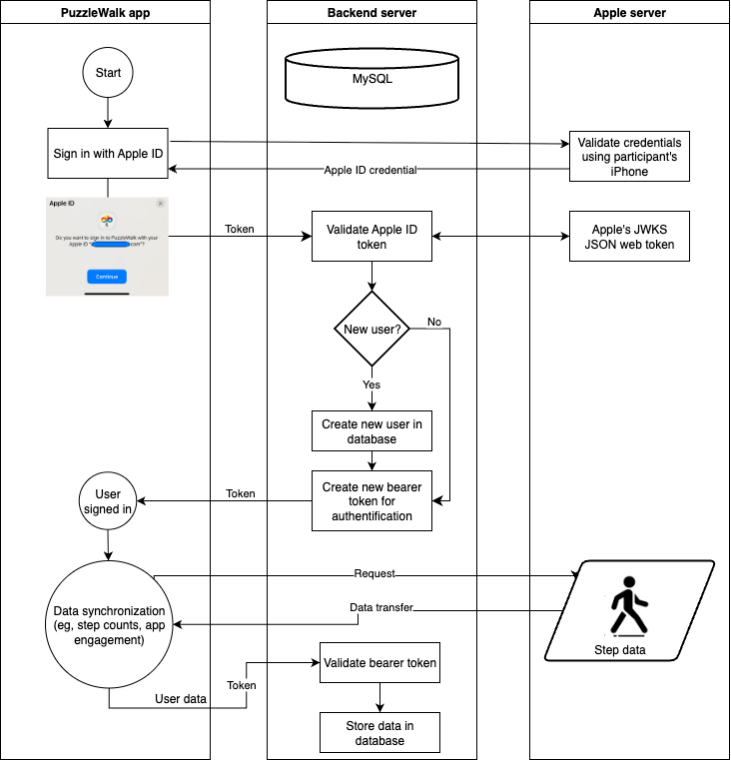
PuzzleWalk system dataflow. JSON: JavaScript Object Notation; JWK: JSON Web Key.

The level of user engagement in the PuzzleWalk intervention will be measured through a Laravel-based backend system, which uses the Voyager administration system for data visualization and management (eg, frequency and duration of app use, step counts, and puzzle points). Time spent on the app in a minutes-per-day format will be calculated to represent each participant’s level of user engagement. These app engagement metrics will be aligned with accelerometer-derived PA and sedentary time to explore how engagement levels relate to daily behavior patterns. Although the study does not include a control group, a 7-day baseline measure will enable within-subject comparison over time. Additional sensitivity analyses will examine how variations in app engagement influence behavioral outcomes during the intervention period. Only activities related to the PuzzleWalk app will be accessed by the software developer and corresponding author as a principal investigator. Any identifiable information, such as email address, will be destroyed immediately upon completion of data collection to ensure participant confidentiality. Follow-up interviews will be conducted after the intervention ends to explore participants’ perceived acceptability, individual experiences (eg, satisfaction challenges), and factors that may have contributed to (dis-)continuation of the app use during and beyond the study. To secure and facilitate dataflow in the PuzzleWalk system, the user authentication process using Apple ID (ie, account credentials) will involve several key steps. First, the user initiates authentication through their Apple ID on the PuzzleWalk app. The app then obtains an authentication token from Apple, and forwards it to the backend server for verification with Apple’s authentication system. Once the token is verified, the backend server generates a session token, enabling the app to access the server application programming interface (API). This session token will facilitate secure API communication, allowing the backend server to interact with the database in real time and manage user requests efficiently (see Figure 3).

### Data Analysis

Accelerometry data will be systematically analyzed by inspecting ranges and addressing missing values in accordance with the previously outlined validation protocol. The raw accelerometry output will be transformed into CPM values and processed using ActiLife software (ActiGraph). To account for the typical lifespan of mHealth interventions (eg, ≤3 months) [35], an intention-to-treat approach will be applied to ensure external validity. Baseline comparisons between autistic adults with and without mild intellectual disability, as well as between male and female autistic adults, will be conducted using independent *t* tests and chi-square tests. We will perform repeated measures ANCOVA to examine the changes in participants’ objective levels of PA and sedentary time before, during, and after the 6-week intervention, adjusting for baseline characteristics, including age, sex, mild intellectual disability, and BMI. Bonferroni post hoc tests will be performed if a significant difference is found between time points. Dependent variables will include light PA, MVPA, sedentary time (minutes per day), MVPA in bouts of >10 minutes per day, steps per day, and user engagement (eg, number of app login sessions and time spent on app use per day). Independent variables will be each time point and intervention. Pearson correlation analyses will be used to examine the relationship between the levels of PA, sedentary time, and user engagement during the intervention period. We will perform data analyses using SPSS v29 (IBM Corp), and the significance level will be set at *P*<.05 (2-tailed).

## Results

Phase I of the project, community-based participatory design workshops (n=9; 4/9, 44% women) was recently concluded. The findings revealed that key autism stakeholders highly valued the app’s self-monitoring and step tracking feature, leaderboard, and visual design. These features were perceived to enhance their perceived motivation for PA engagement and fostered a sense of friendly competition among users. Most stakeholders indicated that the app helped increase PA, reduce SB, and provide a sense of achievement. Autistic traits such as perfectionism and competitiveness reportedly influenced the level of engagement, sometimes enhancing motivation but occasionally causing discouragement due to the difficulty of the puzzle games. Overall, the app was viewed as a potentially beneficial tool for promoting PA and reducing SB in autistic adults (see Table 2). Data collection for Phase II of the project, which involves field deployment (approximate n=70), is currently underway and is expected to be completed in August 2025. As of July 2025, we enrolled 69 participants in the study. The results of this study will be shared in a subsequent publication.

**Table 2:** Key findings of community-based participatory design workshops.

Activities	Key findings
Screening interviews and general inquiry on demographics, patterns of PA^a^ and SB^b^, and overall health among community stakeholders with autism	Most autistic collaborators were diagnosed with ASD^c^ during their early childhood by a medical professional and preferred communication via text messaging or email. Walking was the most preferred form of PA participation among collaborators with autism, but its frequency varied from “rarely” to “four times a week.”
Exploration of preferences and patterns of general technology use and its influence on PA and SB among autistic adults	Health-related mobile apps were generally seen as frustrating or unengaging due to complex interfaces or a lack of engaging features. Feedback highlighted the need for more intuitive functionality and fewer notifications. When it comes to gaming mobile apps, collaborators with autism tended to prefer simple or classic games rather than complex or time-intensive ones. Most collaborators with autism used their smartphones at home or work and typically kept them in a pocket when walking.
Identification of factors influencing PA participation and perceived health benefits of PA	Motivation factors for PA among collaborators with autism varied, ranging from social support provided by family members or therapists to using PA as a means to reduce stress and anxiety. Perceived barriers included low stamina, preference for sedentary activities (eg, watching TV or using social media), and mental health challenges or overthinking. Most collaborators acknowledged the health benefits of PA, particularly its positive effects on physical and mental well-being, as most indicated that they felt happier, healthier, and more fulfilled after engaging in PA.
Evaluation of the core features and identification of areas for improvement in PuzzleWalk v1	Several collaborators suggested adding more puzzle levels and variety to keep the app more engaging. Overall concept (eg, taking steps to play puzzle games) and competitive aspects of the app (eg, leaderboard) were viewed as strengths. Few collaborators recommended incorporating quest-based milestones for unlocking puzzles and intuitive reward systems to motivate continued PA engagement.
Autism-friendly intervention design and pilot compatibility and functionality testing of PuzzleWalk v2 in real-world settings	All 5 collaborators who owned an iPhone during the study successfully installed and tested the app on their devices without any functional issues. Although collaborators appreciated the reward-based system, leaderboard, and step tracking features, 2 of them found the puzzles too challenging, which reduced their perceived enjoyment and continuation desire. According to user feedback, collaborators felt the app effectively promoted self-monitoring of PA and provided a sense of community (eg, competing with other autistic users on the leaderboard) and motivation to be more active during the testing period.

^a^PA: physical activity.

^b^SB: sedentary behavior.

^c^ASD: autism spectrum disorder.

## Discussion

This paper outlines the research protocol for developing and field-testing PuzzleWalk v2, a novel mHealth system designed to promote regular PA and reduce SB in autistic adults with and without mild intellectual disability. The system uses gamification and evidence-based behavior change strategies developed with key autism stakeholders. The protocol establishes a structured methodology for the co-design process and remote deployment of a gamified mHealth intervention that can be scalable in real-world settings. By incorporating community input into the app’s refinement and using replicable, remote data collection methods, this study is positioned to yield critical insights into behavior change strategies and underlying mechanisms among autistic adults. The detailed procedures and supporting resources provided in this paper lay a strong foundation for future evaluation and broader dissemination of tailored digital health interventions for individuals with ASD. It is anticipated that high user engagement with PuzzleWalk v2 will be associated with increased MVPA, higher step counts, and reduced sedentary time among participants with ASD in Phase II.

It is important to note that the vast majority of autism research has focused on understanding the pathology, etiology, and symptoms of this condition with little attention to helping autistic adults live healthy lifestyles [36]. There are limited data on the preventive health behaviors of autistic adults measured by objective and reliable wearable devices in a real-world context [11]. Previous findings on objectively measured PA and SB in individuals with autism have been interpreted with caution because of the small sample sizes with moderate-to-low statistical power [11]. Due to the lack of dedicated surveillance systems, autistic adults who live semi-independently, with and without mild intellectual disability, have not been precisely monitored, as they are mostly not affiliated with community organizations or public school systems [3]. Identifying effective recruitment strategies to upscale the sample size and verifying the feasibility of remote intervention delivery methods are major strengths of this protocol.

Given the prevalent social and sensory challenges, traditional face-to-face PA interventions in community-based public settings may not be a sustainable behavior change model nor an effective strategy for autistic adults [37,38]. Due to the complexities of the intervention delivery and assessment protocols, the existing evidence is insufficient to confirm that traditional exercise interventions lead to long-term improvements in PA levels in this population [11]. Although the benefits of regular, face-to-face PA participation are well-documented, it is critical for researchers, educators, and service providers to diversify the PA options for autistic adults by creating environments that are inclusive, relevant, and sensitive to their needs and preferences [15,39]. While mobile technology research has made significant progress in improving the life skills of autistic individuals (eg, social and communication skills) [40], only a few technological attempts have been made to address PA engagement issues in this population. Gamification strategies have emerged as a viable approach for enhancing PA levels and reducing sitting time, especially in those with low intrinsic motivation for voluntary behavior changes [19,41]. However, research on the practicality of this gamification approach in mHealth interventions remains in its early stages and is not yet fully understood for autistic adults, who may have unique preferences, motivational factors, and barriers to behavior change [15]. In addition, mHealth-guided behavior change interventions generally result in only short-term participant engagement [42]. Rigorous empirical research is essential to develop replicable and comprehensive interventions grounded in evidence-based frameworks and to promote sustainable PA behavior changes for autistic adults [15].

Historically, autism research has often misrepresented the autism community by excluding individuals with ASD from the research process, using outdated or stigmatizing language, and prioritizing research agendas that may not align with autism community values [25,43]. In response to the increasing demand for collaborative autism health research, this study used community-based workshops to identify achievable strategies for mHealth-guided PA and SB changes. Subsequently, these workshops also informed the gamification design aesthetics for tailoring the intervention to the needs of autistic adults. To establish alignment between the research team and key autism stakeholders, we leveraged the practical guidelines for collaborative autism research, informed by AASPIRE [25]. The results of the field deployment study (Phase II) will be shared with the workshop participants, and their additional input will be sought to refine the intervention protocol for future clinical trials with a control group (eg, longitudinal randomized controlled trials).

A limitation of this study is the restriction of eligibility to iPhone users, which may introduce sampling bias. Although an Android version of the app is currently in development, this criterion may exclude individuals who cannot afford iPhones or prefer Android devices. Consequently, a significant portion of autistic adults, particularly those with financial constraints or differing technology preferences, may be underrepresented. Therefore, future work should focus on expanding and evaluating PuzzleWalk v2 across a wide range of smartphone brands and models to enhance accessibility and scalability. Given that individuals who choose to participate in this game-oriented mHealth study may have a greater interest in or familiarity with mobile gaming, the use of a robust recruitment strategy and inclusion of a diverse and adequately powered sample are expected to reduce sampling bias and to enhance the generalizability of the findings. Study results will be disseminated through various channels, including scholarly conference presentations and peer-reviewed publications. Furthermore, findings from this study will guide the continued refinement of the PuzzleWalk system, with the long-term goal of facilitating its future public release and broader implementation. Future research should also explore the influence of community-level factors on the successful implementation of PA intervention strategies. Given the importance of ecological validity in PA research [44], community-level factors, including associability and affordability of community resources, social support networks, and environmental conditions that facilitate PA participation, should be considered in designing and evaluating PA interventions for autistic adults.

### Conclusions

The PuzzleWalk app demonstrates its usability as an innovative tool for enhancing both PA motivation and intervention engagement through tailored gamification and behavior change strategies. The results of our ongoing field deployment study will clarify the app’s effectiveness and real-world applicability in promoting PA and reducing SB in autistic adults.

## Appendix

Multimedia Appendix 1 Multi-format Co-Design Workshop Guide.

Multimedia Appendix 2 Monitor wear and return guidelines.

Multimedia Appendix 3 Peer review report by the Autism Intervention Research Network on Physical Health and the University of Delaware’s Maggie E. Neumann Health Sciences Research Fund.

Multimedia Appendix 4 SPIRIT Checklist.

## Abbreviations

AASPIRE: Academic Autistic Spectrum Partnership in Research and Education
API: application programming interface
ASD: autism spectrum disorder
CPM: counts per minute
mHealth: mobile health
MVPA: moderate-to-vigorous physical activity
PA: physical activity
SB: sedentary behavior
SPARK: Simons Foundation Powering Autism Research for Knowledge
