# Supplementary material for: A Gamified mHealth App to Promote Physical Activity and Reduce Sedentary Behavior in Autistic Adults: Protocol for a Remotely Delivered Pilot Intervention Study
Source: JMIR Res Protoc. 2025 Jul 22;14:e71631. doi: 10.2196/71631 (PMC12326157; doi:10.2196/71631)
Supplement: Multimedia Appendix 1 [file resprot_v14i1e71631_app1.docx]

**Multi-format Co-Design Workshop Guide**

This topic guide summarizes the core questions and discussion prompts used throughout the participatory design phase with autistic stakeholders. The goal was to explore behavioral patterns, preferences, and user experiences to inform the iterative refinement of the PuzzleWalk app.

| **Timeline** | **Topics for discussion** |
| --- | --- |
| **Week 1** | 1. **Initial screening and health background**  - Demographics - Autism-related information - Health status and medical history - Physical activity and sedentary behavior patterns |
| **Week 2** | 1. **General technology use and preferences**  - What kind of IT devices do you use regularly (e.g., smartphone, tablet, desktop)? - Where do you primarily use these devices (home, work, school)? - What type of smartphone do you use (iPhone/Android)? - What apps do you use for: a) Social networking, b) Reading and information seeking, c) Communication, d) Entertainment (e.g., games, videos, music), f) Health or fitness tracking - How much time do you spend on your phone during weekdays and weekends? - How do you feel when using your smartphone? - How do you feel when you can't use it? - What health or fitness apps do you currently use? - What do you like/dislike about health or fitness apps? |
| **Week 3-4** | 1. **Physical activity participation**  - What does being physically active mean to you? - What are your favorite and least favorite physical activities, and why? - How do these activities make you feel? - How active are you on weekdays and weekends? - Do you prefer individual or team-based activities, and why? - What motivates or prevents you from being physically active? - How has your physical activity level changed over time? - What are the things that help you participate in physical activities? - What do you do that has a positive impact on your health? - Do you think that physical activity is important for your health? Please explain. |

| **Week 5-6** | 1. **PuzzleWalk v1 Evaluation**   *Before completing the app evaluation, participants were asked to review the PuzzleWalk User Guide and watch a brief step-by-step introductory video. These materials provided a visual and conceptual overview of the app’s core features, such as gamified step-to-game conversion and puzzle games. This aimed to ensure participants had a clear understanding of the app’s intended use prior to providing feedback.*   - What are the core features of the PuzzleWalk app? - Which features did you find most engaging or interesting? - What elements of the app could be improved? - How can we improve the app’s ability to engage autistic adults? - How can we increase user engagement to support long-term improvements in physical activity? |
| --- | --- |
| ***Integration of key findings into PuzzleWalk v2 over three months*** | |
| **Week 7-8** | 1. **Field testing of PuzzleWalk v2**   *Five stakeholders freely tested the PuzzleWalk v2 app in real-world settings over a period of up to two weeks. This activity focused on evaluating autism-friendly intervention design, as well as pilot testing the app’s compatibility and functionality. Participant feedback was used to fine-tune the app’s core features and assess its potential for promoting physical activity among autistic adults in real-world settings prior to broader deployment.*   - What app features did you enjoy the most? - What factors reduced your enjoyment (e.g., glitches, difficulty)? - What benefits did the app provide in terms of motivation for behavior change? - Were there any features you felt were missing? - Did the app help increase your physical activity and reduce sedentary behavior? - How might autistic traits affect the use of the app? |
