# Supplementary material for: A Gamified mHealth App to Promote Physical Activity and Reduce Sedentary Behavior in Autistic Adults: Protocol for a Remotely Delivered Pilot Intervention Study
Source: JMIR Res Protoc. 2025 Jul 22;14:e71631. doi: 10.2196/71631 (PMC12326157; doi:10.2196/71631)
Supplement: Multimedia Appendix 2 [file resprot_v14i1e71631_app2.pdf]

Monitor Wear Guideline (Weeks 1, 3, 5, and 8)

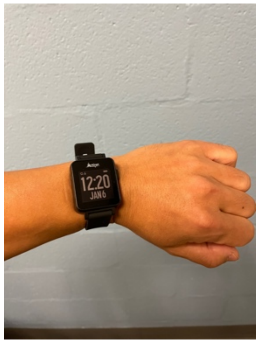	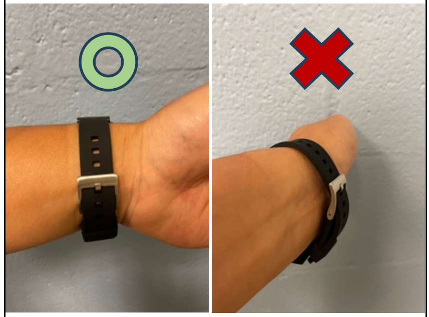	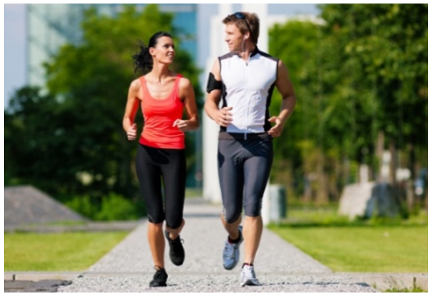	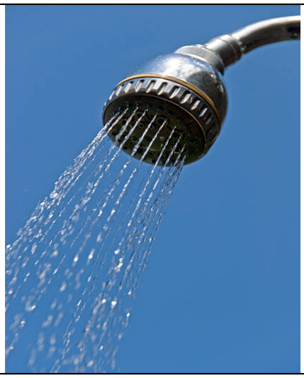
1. Wear the smartwatch on your <u>**non-dominant wrist.**</u>	2. Place the watch high up on your wrist with appropriate tightness.	3. Carry out your normal activities of daily living during waking hours.	4. To avoid water damage, take off the watch and put it in a safe place when you take a shower or swim in the pool.
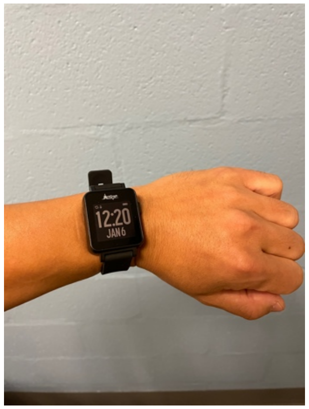	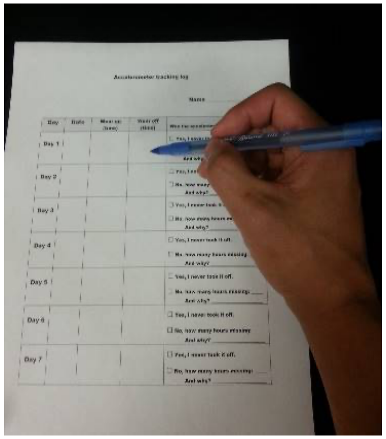	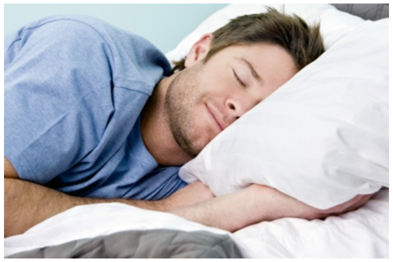	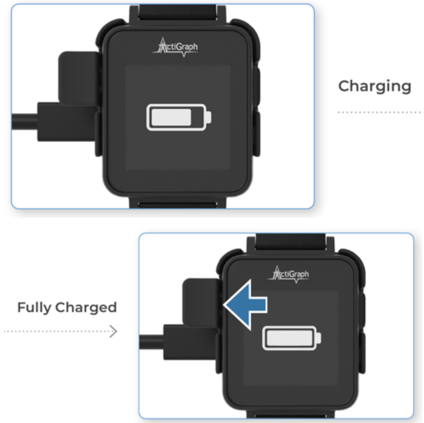
5. Wear the watch again when you finish taking a shower or swimming.	6. Right before going to bed, fill in your monitor tracking log (wear on/off time).	7. Take off your watch and put it in a safe place when you go to bed. When you wake up, wear the watch again and enjoy your day!	**Charging* is required only once every three weeks. Our research team will remind you of the times that you need to charge the watch (up to 3 hours for full charge).

***Please contact the research team if you have any questions.**

University of Delaware Physical Activity and Technology for All Lab (puzzlewalk.acc@gmail.com)

GUIDE TO RETURN YOUR SMARTWATCH AND CHARGER

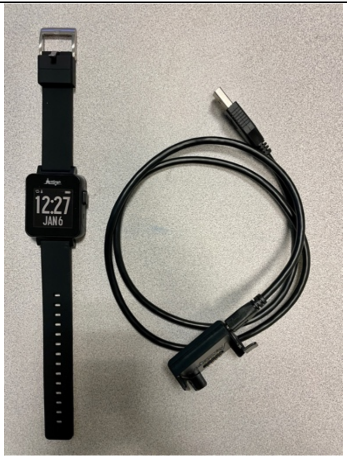	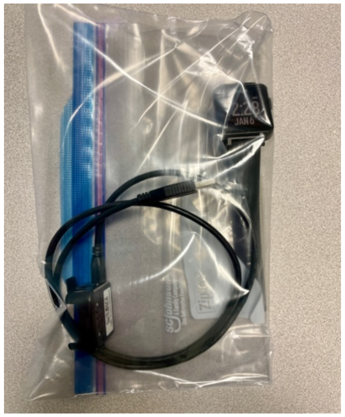	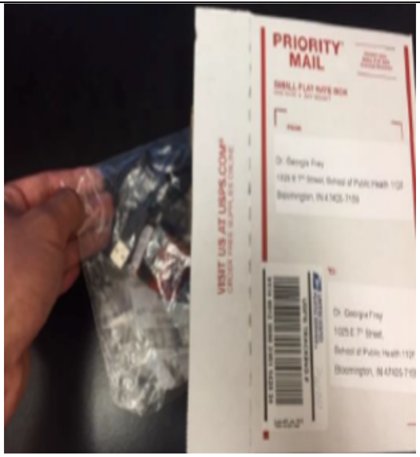	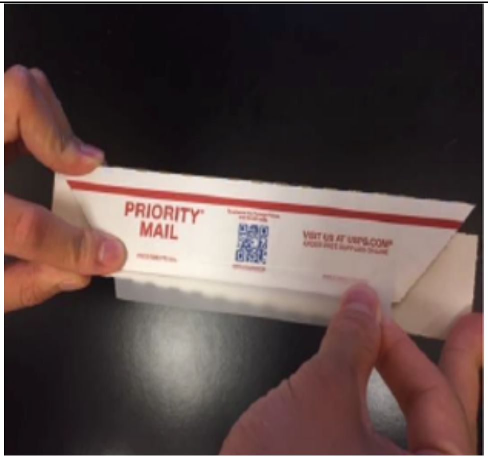
1. Prepare your smartwatch, charger (a charging dock with cable), and monitor tracking logs.	2. Put all your study materials in the provided Ziplock bag.	3. Put the Ziplock bag containing all the study materials into the provided Priority Mailbox.	4. Seal the box using adhesive tape (**Please seal tight!**).
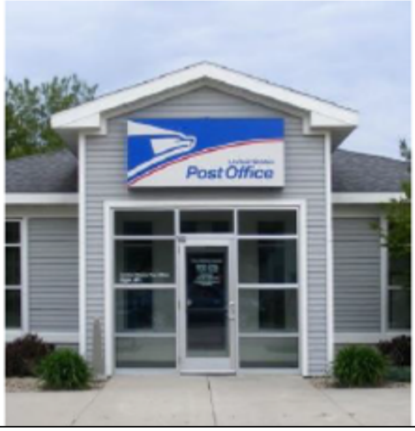	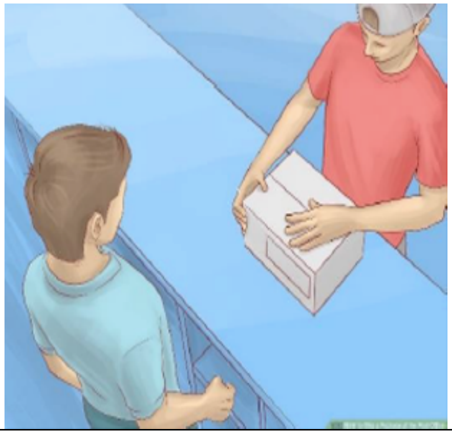	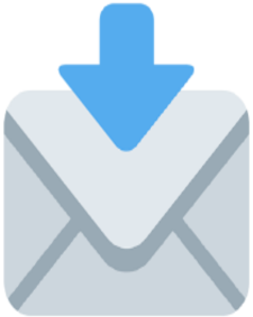	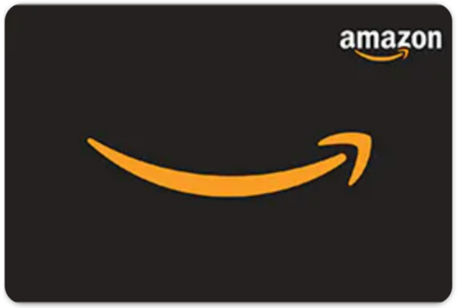
5. Go to the USPS post office near your location.	6. Mail the self-addressed, prepaid Priority Mailbox to the research team.	7. Wait for a confirmation email from the research team.	8. Once your study compliance is verified (e.g., watch wear, survey participation), we will send you the $100 Amazon e-gift card via email.

**The Priority Mailbox is self-addressed and prepaid, so you do NOT need to purchase a stamp for returning!*

**Mail recipient: Dr. Daehyoung Lee – 013 Carpenter Sports Building, 26 N College Avenue, Newark, DE 19716*
