# Supplementary material for: A Gamified mHealth App to Promote Physical Activity and Reduce Sedentary Behavior in Autistic Adults: Protocol for a Remotely Delivered Pilot Intervention Study
Source: JMIR Res Protoc. 2025 Jul 22;14:e71631. doi: 10.2196/71631 (PMC12326157; doi:10.2196/71631)
Supplement: Multimedia Appendix 3 [file resprot_v14i1e71631_app3.pdf]

AUTISM INTERVENTION RESEARCH NETWORK ON PHYSICAL HEALTH

Reviewer 1:

- The researchers are proposing to evaluate the feasibility and efficacy of a mobile App intervention for improving physical activity, and reducing sedentary time, among adults with ASD.
- Strong justification is provided for this study's need, given evidence for low engagement in these health behaviors, and associated high health risk, within this population.
- Furthermore, strong justification is provided for why a mobile app intervention is appropriate and likely to be successful among adults with ASD. The design of the mobile app is evidence- and theory- based, and as such it offers an innovative approach to health promotion within this population.
- The app was previously developed and tested by the PI of the present study. Prior evaluations indicated positive effects on health behaviors, but only for the first 4-weeks of the 8-week intervention.
- This study is seeking to engage autistic stakeholders in participatory workshops to improve autism-friendly features in effort to improve use and impact of the intervention among adults with ASD. The engagement of multiple stakeholders, including self-advocates, parents of adults with autism, and autistic scholars and app designers, is a strength of the proposal. Clear community participation guidelines are provided that suggest researchers were intentional in their efforts to facilitate equitable contributions from diverse team members (as is reflected in equal pay, and distribution of decision making and leadership roles).
- The secondary pre-post study design is well-defined with appropriate measures and analysis selected, and a well-justified sample size. The study is limited in the recruitment efforts to involve a under-represented and marginalized groups across race, sex or disability. However, researchers indicated clear efforts to support participants with mild intellectual disabilities, with their methods well-aligned with best-practice recommendations.
- Given that this app has been previously tested, seems a missed opportunity to not explore in more depth why participants disengage with the app, or continue to see behavior change benefits after 4-weeks. Alternatively, alongside the participatory workshops, a comparative study of version 1 and version 2 may help identify which features are most effective in sustaining target health behaviors or motivating continued participation. These are just ideas to encourage researchers to seek depth in their App evaluation.
- Overall, the research team and environment is well equipped to conduct this research.

Reviewer 2:

The study addresses an important problem concerning physical activity (PA) and sedentary behavior (SB) in autistic adults, emphasizing the limited research in this area and the potential health risks associated with prolonged sitting. The project aims to use a gamified mobile app called PuzzleWalk v2 as an intervention to increase PA and reduce SB in this population. It incorporates participatory design workshops involving community stakeholders, including autistic individuals, to optimize the app's design decisions and incorporate autism-friendly elements.

The research design, using a pre-/post-test design with Repeated Measures Analysis of Variance, focuses on examining changes in participants' objective levels of PA and sedentary time before, during, and after the 8-week intervention. However, the absence of a control group limits the ability to compare the intervention's effectiveness with other similar interventions. Additionally, the lack of long-term follow-up to assess the sustainability of behavior change induced by the app is mentioned, raising concerns about the app's long-term impact.

The project incorporates several novel concepts, approaches, and methods, including the use of gamified mHealth interventions, a conversion algorithm for step counts, and participatory design workshops. The research environment, including the College of Health Sciences and the Science, Technology and Advanced Research (STAR) Campus at the University of Delaware, provides a conducive setting for the project, supported by the Center for Human Research Coordination and the Biostatistics Core.

The review also addresses the project's inclusion of under-represented and marginalized groups, emphasizing the need to diversify recruitment efforts and minimize implicit bias. While the project acknowledges the importance of diversity, specific strategies for inclusion are not explicitly outlined. The review highlights the importance of considering alternative tactics to mitigate potential biases and confounding factors.

Overall, the proposed research project demonstrates a commitment to addressing the physical health needs of autistic adults through innovative interventions. However, there are areas for improvement, such as incorporating a control group, implementing long-term follow-up assessments, and providing more detailed plans for the inclusion of under-represented and marginalized groups. These revisions would strengthen the study's design, comparability, and generalizability, contributing to a more robust understanding of the intervention's effectiveness and sustainability.

MAGGIE E NEUMANN FUND
Review

Principal Investigator(s): Daehyoung Lee

OVERALL IMPACT

Reviewers will provide an overall impact score to reflect their assessment of the likelihood for the interdisciplinary project to exert a clear proof-of-concept for novel and potentially scalable approaches to improve the health and well-being of those with chronic disabilities, in consideration of the following five scored review criteria, and additional review criteria. Scoring will be on a 9-point scale where 1= Exceptional and 9= Poor. An application does not need to be strong in all categories to be judged likely to have major scientific impact.

SCORED REVIEW CRITERIA

Overall Impact Score [1-9]: 3

Innovative approach to addressing health promotion among population of autistic adults. There is clear involvement of the target population in the development of the intervention and its implementation. The project team brings different areas of expertise to the study, although it is not clear if this is an established team or one just formed for this project. Past experience in recruiting a small sample is promising.

Reviewers will consider each of the five review criteria below in the determination of scientific and technical merit and give a separate score for each.

1. Significance

Significance score [1-9]: 3

Does the project address an important problem or a critical barrier to progress in the field? If the aims of the project are achieved, how will the scientific knowledge, technical capability, and/or clinical practices improve the health and/or quality of life for adults or children in Delaware with chronic disabilities? How will successful completion of the aims change the concepts, methods, technologies, treatments, services, or preventative interventions that drive this field?

Proposes to test feasibility & acceptability of gamified mobile health intervention to increase PA & reduce SB among autisitic adults w/ & w/o mild intellectual disability. Specific aims focus on identifying barriers to mHealth-based changes in PA/SB, and evaluate feasibility & acceptability of PuzzleWalk (PW) intervention.

Strengths

- Identifies and addresses a significant health behavior need in the population. Design is consistent with identified preferences of the population

Weaknesses

- Will 8 weeks be long enough to produce persistence (a noted weakness identified in other approaches)? Noted in small pilot that took 5 weeks to establish persistence.

Sample is relatively small (n=36)

2. Investigator(s) and Environment [1-9]: 4

Are the PD/PIs, collaborators, and other researchers interdisciplinary (i.e., hold expertise in distinct fields/areas) and well suited to the project? If Post-Doc is the named PI, is a suitable mentor listed and mentoring plan briefly described? Do investigators have a demonstrated track-record working with the targeted community and relevant community/commercial partners? If the project is collaborative or multi-PD/PI, do the investigators have complementary and integrated expertise; are their leadership approach, governance, and organizational structure appropriate f or the project, and sufficiently inclusive? Does the application have sufficient access to facilities and resources to carry out the proposal?

Strengths

- Team includes members with expertise in both working with autistic adults as well as in human computer interactions. Members represent distinct disciplinary approaches and roles are well defined.

Weaknesses

- Team appears to be somewhat newly formed as none of biosketches point to existing collaboration.

3. Innovation [1-9]: 3

Does the application challenge and seek to shift current research or clinical practice paradigms by utilizing novel theoretical concepts, approaches or methodologies, instrumentation, or interventions? Will the results of this study directly inform and support a larger subsequent grant application that will improve the health and/or quality of life for adults and/or children with disabilities in Delaware?

Strengths

- Approach represents an innovative mHealth approach. Use of CPBR techniques enhance refinement and likelihood for acceptability of intervention. recruitment model is identified as innovative and proposes to work with multiple entities supporting autistic adults. Identifies an appropriate (NIDILRR) funding mechanism that this project can inform.

Weaknesses

Greater articulation of past successes in recruiting the target population would strengthen.

4. Approach [1-9]: 4

Are the overall strategy, methodology, and analyses well-reasoned and appropriate to accomplish the specific aims of the project? Are community partners sufficiently engaged? Is the study design justified and feasible? Is the sample size sufficient? Are the targeted population and study setting appropriate for the proposed research question(s)? Are the assessments validated and appropriate for the study population?

Strengths

- Participatory design workshop is clear and likely to provide valuable information to enhance feasibility and acceptability of intervention.

Weaknesses

- Although accelerometry can be successfully used to capture PA, it's not a true measure

of SB, but can capture inactivity.

5. Target Population-centeredness and stakeholder engagement [1-9]:

Does the application include a description of how the outcomes are important to the target population? Is the engagement of relevant target groups and other stakeholders described? Are the strategies to include targeted communities appropriate to the study?

Strengths

- Clear identification of outcomes related to health needs of the population. Strong involvement of ultimate users (autistic adults)

Weaknesses

ADDITIONAL REVIEW CONSIDERATIONS

As applicable for the project proposed, reviewers will address each of the following items, but will not give scores for these items and should not consider them in providing an overall impact/priority score.

Principal Investigator or Co-Investigator is a current faculty member of the College of Health Sciences
Comments (if applicable):<br>• Yes

Letters of support are provided.
Comments (if applicable):<br>• Strong letters of support from relevant sources for recruitment.

Budget is for 12-months, falls between $25,000 - $55,000, and meets requirements
Comments (if applicable):<br>• Yes

Timeline
Comments (if applicable):<br>• Appears feasible

Explanation of Scoring

Impact	Score	Descriptor	Additional Guidance on Strengths/Weaknesses
High	1	Exceptional	Exceptionally strong with essentially no weaknesses
	2	Outstanding	Extremely strong with negligible weaknesses
	3	Excellent	Very strong with only some minor weaknesses
Medium	4	Very Good	Strong but with numerous minor weaknesses
	5	Good	Strong but with at least one moderate weakness
	6	Satisfactory	Some strengths but also some moderate weaknesses
Low	7	Fair	Some strengths but with at least one major weakness
	8	Marginal	A few strengths and a few major weaknesses
	9	Poor	Very few strengths and numerous major weaknesses
**Non-numeric score options:** NR = Not Recommended for Further Consideration, DF = Deferred, AB = Abstention, CF = Conflict, NP = Not Present, ND = Not Discussed			
**Minor Weakness:** An easily addressable weakness that does not substantially lessen impact<br>**Moderate Weakness:** A weakness that lessens impact<br>**Major Weakness:** A weakness that severely limits impact			

MAGGIE E NEUMANN FUND
Review

Principal Investigator(s): ***Lee, Daehyoung***

OVERALL IMPACT

Reviewers will provide an overall impact score to reflect their assessment of the likelihood for the interdisciplinary project to exert a clear proof-of-concept for novel and potentially scalable approaches to improve the health and well-being of those with chronic disabilities, in consideration of the following five scored review criteria, and additional review criteria. Scoring will be on a 9-point scale where 1= Exceptional and 9= Poor. An application does not need to be strong in all categories to be judged likely to have major scientific impact.

Overall Impact Score [1-9]:
Write a paragraph summarizing the factors that informed your Overall Impact score.

Overall Impact score 1
This proposal directly addresses a significant health disparity by aiming to develop and test a gamified mobile health (mHealth) app called PuzzleWalk to increase physical activity and reduce sedentary behavior in adults with autism spectrum disorder (ASD) and intellectual disabilities (ID). The innovative approach leverages robust community-based participatory research methods by involving the autism community and interdisciplinary collaborators throughout the research process. The proposal aligns with the funding Neumann fund's mission to improve health and well-being, as it provides a pathway for the autism community to directly engage in the research. The experienced investigative team, led by the PI's expertise in mHealth interventions, has a proven track record of securing federal funding and publishing in high-impact journals. With a rigorous methodology, appropriate power calculation, and a suitable analytical plan, this proposal demonstrates a high likelihood of generating meaningful data to support future research and interventions tailored for underserved populations. Overall, this exceptional proposal comprehensively addresses all review criteria outlined by the Maggie E. Neumann Health Sciences Research Fund.

SCORED REVIEW CRITERIA

Reviewers will consider each of the five review criteria below in the determination of scientific and technical merit, and give a separate score for each.

1. Significance score [1-9]:

Does the project address an important problem or a critical barrier to progress in the field? If the aims of the project are achieved, how will the scientific knowledge, technical capability, and/or clinical practices improve the health and/or quality of life for adults or children in Delaware with chronic disabilities? How will successful completion of the aims change the concepts, methods, technologies, treatments, services, or preventative interventions that drive this field?

Score 1

Strengths

- *The proposal directly addresses a significant health disparity of low physical activity and high sedentary behavior in adults with ASD and intellectual disabilities (ID), which aligns with national priorities.*
- *The background information clearly establishes the prevalence, health risks, and unique challenges faced by this underserved population.*

Weaknesses

☐None

2. Investigator(s) and Environment [1-9]:

Are the PD/PIs, collaborators, and other researchers interdisciplinary (i.e., hold expertise in distinct fields/areas) and well suited to the project? If Post-Doc is the named PI, is a suitable mentor listed and mentoring plan briefly described? Do investigators have a demonstrated track-record working with the targeted community and relevant community/commercial partners? If the project is collaborative or multi-PD/PI, do the investigators have complementary and integrated expertise; are their leadership approach, governance, and organizational structure appropriate for the project, and sufficiently inclusive? Does the application have sufficient access to facilities and resources to carry out the proposal?

Score 1

Strengths

- *Dr. Lee has an excellent track record of publications, funding, and expertise in mHealth interventions, particularly for autism spectrum disorder (ASD), as evidenced by his biosketch.*
- *The co-investigator, Dr. Anjana Bhat, is also highly accomplished in autism research with extensive funding and mentorship experience at the University of Delaware, providing a strong environment for this interdisciplinary project.*

Weaknesses

None

3. Innovation [1-9]:

Does the application challenge and seek to shift current research or clinical practice paradigms by utilizing novel theoretical concepts, approaches or methodologies, instrumentation, or interventions? Will the results of this study directly inform and support a larger subsequent grant application that will improve the health and/or quality of life for adults and/or children with disabilities in Delaware?

Score 1

Strengths

- *Highly innovative approach adapting gamification and behavior change techniques for a tailored mHealth app for autistic adults*
- *Novel use of participatory design workshops with the autism community*
- *Interdisciplinary team includes an autistic software developer, enhancing innovation*

Weaknesses

- None noted

4. Approach [1-9]:

Are the overall strategy, methodology, and analyses well-reasoned and appropriate to accomplish the specific aims of the project? Are community partners sufficiently engaged? Is the study design justified and feasible? Is the sample size sufficient? Are the targeted population and study setting appropriate for the proposed research question(s)? Are the assessments validated and appropriate for the study population?

Score 1

Strengths

- *The community-based participatory research (CBPR) approach, involving key stakeholders like autistic adults, caregivers, and therapists throughout the research process, is a major strength.*
- *The multi-source recruitment strategy leveraging autism organizations and online support groups is robust and likely to achieve the required sample size.*
- *The use of participatory design workshops and iterative development process increases the likelihood of an acceptable and feasible intervention.*
- *Appropriate sample size calculation, eligibility criteria, and objective physical activity/sedentary time measurement methods are described.*

Weaknesses: *None noted*

5. Target Population-centeredness and stakeholder engagement [1-9]:

Does the application include a description of how the outcomes are important to the target population? Is the engagement of relevant target groups and other stakeholders described? Are the strategies to include targeted communities appropriate to the study?

Score 1

Strengths

- *The proposal demonstrates a deep understanding of the target population's unique needs and challenges.*
- *The CBPR approach and alignment with Autism Speaks' research priorities ensure the intervention is tailored and acceptable to autistic adults.*
- *There is a strong plan for engaging the autism community throughout the research process and disseminating findings to stakeholders.*

ADDITIONAL REVIEW CONSIDERATIONS

As applicable for the project proposed, reviewers will address each of the following items, but will not give scores for these items and should not consider them in providing an overall impact/priority score.

Principal Investigator or Co-Investigator is a current faculty member of the College of Health Sciences
Comments (if applicable):<br>*The Principal Investigator is Dr. Daehyoung Lee, a faculty member in the Department of Health Behavior and Nutrition Sciences at the University of Delaware, which is part of the College of Health Sciences. This meets the eligibility requirement.*

Letters of support are provided.
Comments (if applicable):<br>*Support Letters from Directors of Autism Delaware and Autism awareness organization are provided*

Budget is for 12-months, falls between $25,000 - $50,000, and meets requirements
Comments (if applicable):<br>*The application is budgeted at $52,145 (maximum allowed $55,000).*

Timeline
Comments (if applicable):<br>*The work should be able to be finished in the 12 month timeline allowed as per my opinion.*<br>*Please note, however, that I did not find an explicit statement about adhering to that timeline in the provided proposal excerpts.*

Explanation of Scoring

Impact	Score	Descriptor	Additional Guidance on Strengths/Weaknesses
High	1	Exceptional	Exceptionally strong with essentially no weaknesses
	2	Outstanding	Extremely strong with negligible weaknesses
	3	Excellent	Very strong with only some minor weaknesses
Medium	4	Very Good	Strong but with numerous minor weaknesses
	5	Good	Strong but with at least one moderate weakness
	6	Satisfactory	Some strengths but also some moderate weaknesses
Low	7	Fair	Some strengths but with at least one major weakness
	8	Marginal	A few strengths and a few major weaknesses
	9	Poor	Very few strengths and numerous major weaknesses

Non-numeric score options: NR = Not Recommended for Further Consideration,
DF = Deferred, AB = Abstention, CF = Conflict, NP = Not Present, ND = Not Discussed

Minor Weakness: An easily addressable weakness that does not substantially lessen impact
Moderate Weakness: A weakness that lessens impact
Major Weakness: A weakness that severely limits impact

MAGGIE E NEUMANN FUND

Review

Principal Investigator(s): Daehyoung Lee PhD

OVERALL IMPACT

Reviewers will provide an overall impact score to reflect their assessment of the likelihood for the interdisciplinary project to exert a clear proof-of-concept for novel and potentially scalable approaches to improve the health and well-being of those with chronic disabilities, in consideration of the following five scored review criteria, and additional review criteria. Scoring will be on a 9-point scale where 1= Exceptional and 9= Poor. An application does not need to be strong in all categories to be judged likely to have major scientific impact.

SCORED REVIEW CRITERIA

Overall Impact Score [1-9]: 3

Write a paragraph summarizing the factors that informed your Overall Impact Score.

A strong interdisciplinary team propose to refine a mHealth intervention to increase physical activity and reduce sedentary time in autistic adolescents. The proposed solution is based on considerable work by the PI based on on-going funding and recent publications. The proposed solution is potentially scalable and effective. The engagement in the target population in the conceptualization and formative testing in a strength and supported by relevant community organization involvement. This study is likely to have a high impact.

Reviewers will consider each of the five review criteria below in the determination of scientific and technical merit and give a separate score for each.

1. Significance

Significance score [1-9]: 5

Does the project address an important problem or a critical barrier to progress in the field? If the aims of the project are achieved, how will the scientific knowledge, technical capability, and/or clinical practices improve the health and/or quality of life for adults or children in Delaware with chronic disabilities? How will successful completion of the aims change the concepts, methods, technologies, treatments, services, or preventative interventions that drive this field?

Strengths

- This proposal meets the Maggie Neumann Research Fund's criteria for innovative, scalable methods to enhance the health of individuals with chronic disabilities.
- High levels of sedentary behavior and low physical activity are common in autistic adults and are key predictors to chronic diseases in this group.

Weaknesses

- Not clear why community collaborators and not end-users/target population are being recruited for aim 1; the results of this formative work may not generalize to end users and so limit the impact/significance of prototype being developed. – Edited to add that in the methods 'community collaborators' was defined to include autistic adults – 'community collaborators' not typically used to

describe study participants

- The premise that there are limited data on device-estimates of health behaviors in autistic adults is somewhat of a sweeping statement and that this is one of the first to use triaxial devices to measure these behaviors in this population is somewhat of an overstatement (minor)
- Limited to no explanation of the actual gamified mHealth intervention, so unable to evaluate potential impact/significance of achieving the aims of this study

2. Investigator(s) and Environment [1-9]: 2

Are the PD/PIs, collaborators, and other researchers interdisciplinary (i.e., hold expertise in distinct fields/areas) and well suited to the project? If Post-Doc is the named PI, is a suitable mentor listed and mentoring plan briefly described? Do investigators have a demonstrated track-record working with the targeted community and relevant community/commercial partners? If the project is collaborative or multi-PD/PI, do the investigators have complementary and integrated expertise; are their leadership approach, governance, and organizational structure appropriate f or the project, and sufficiently inclusive? Does the application have sufficient access to facilities and resources to carry out the proposal.

Strengths

- Strong interdisciplinary investigative team (expertise in movement and disability studies, physical therapy, computer science) with relevant expertise in physical activity and sedentary behavior in adults with disabilities as well as mHealth interventions.
- Dr. Lee has the necessary training and experience to lead this work, including ongoing support from the Autism Intervention Research Network (overlap?).
- Environment is suitable to conduct the work as described.
- Letter of support from the Disability Section chair for the American Public Health Association and Autism Delaware.

Weaknesses

- None noted.

3. Innovation [1-9]: 3

Does the application challenge and seek to shift current research or clinical practice paradigms by utilizing novel theoretical concepts, approaches or methodologies, instrumentation, or interventions? Will the results of this study directly inform and support a larger subsequent grant application that will improve the health and/or quality of life for adults and/or children with disabilities in Delaware?

Strengths

- The use of mHealth to improve health behaviors in autistic adults remains a novel idea that has yet to be fully operationalized and tested. Dr. Lee's proposed approach will make significant in-roads in this regard.
- Plausible future grant applications are detailed in the application.

Weaknesses

- There is very limited description of the actual mHealth intervention; nevertheless the PI has recent publications and ongoing funding suggesting the existence of this intervention.

4. Approach [1-9]: 3

Are the overall strategy, methodology, and analyses well-reasoned and appropriate to accomplish the specific aims of the project? Are community partners sufficiently engaged? Is the study design justified and feasible? Is the sample size sufficient? Are the targeted population and study setting appropriate for the proposed research question(s)? Are the assessments validated and appropriate for the study population?

Strengths

- Actigraph processing protocol is appropriate (although not specifically designed for assessing sedentary behavior per se).
- A clearly reported sample justification for Aim 2.

Weaknesses

- The age range is vast (18-55y) – is an mHealth intervention likely to appeal to mid-life adults? Is it feasible to have an effective intervention that is appealing to such a wide age range? Also, there may be heterogeneity of treatment because of the wide age range.
- What is the target sample size for Aim 1 and how many of each sub-group (autistic adults, parents/caregivers, autism therapists) are expected to get saturation? Number of sessions/meetings not explicitly stated. Analysis methods for this information not described. (later seen in Budget justification)

5. Target Population-centeredness and stakeholder engagement [1-9]: 1

Does the application include a description of how the outcomes are important to the target population? Is the engagement of relevant target groups and other stakeholders described? Are the strategies to include targeted communities appropriate to the study?

Strengths

- A clear and comprehensive plan for engagement with the target community and relevant stakeholders is described. The inclusion of 15 autistic adults in planning and testing of materials is excellent.
- Partnership with Autism Speaks is a notable strength.

Weaknesses

- None noted

ADDITIONAL REVIEW CONSIDERATIONS

As applicable for the project proposed, reviewers will address each of the following items, but will not give scores for these items and should not consider them in providing an overall impact/priority score.

Principal Investigator or Co-Investigator is a current faculty member of the College of Health Sciences
Comments (if applicable):<br>• yes

Timeline
Comments (if applicable):<br>• ok

Letters of support are provided.
Comments (if applicable):<br>• yes

Budget is for 12-months, falls between $25,000 - $55,000, and meets requirements
Comments (if applicable):<br>• yes

Explanation of Scoring

Impact	Score	Descriptor	Additional Guidance on Strengths/Weaknesses
High	1	Exceptional	Exceptionally strong with essentially no weaknesses
	2	Outstanding	Extremely strong with negligible weaknesses
	3	Excellent	Very strong with only some minor weaknesses
Medium	4	Very Good	Strong but with numerous minor weaknesses
	5	Good	Strong but with at least one moderate weakness
	6	Satisfactory	Some strengths but also some moderate weaknesses
Low	7	Fair	Some strengths but with at least one major weakness
	8	Marginal	A few strengths and a few major weaknesses
	9	Poor	Very few strengths and numerous major weaknesses
**Non-numeric score options:** NR = Not Recommended for Further Consideration,<br>DF = Deferred, AB = Abstention, CF = Conflict, NP = Not Present, ND = Not Discussed			
**Minor Weakness:** An easily addressable weakness that does not substantially lessen impact<br>**Moderate Weakness:** A weakness that lessens impact<br>**Major Weakness:** A weakness that severely limits impact			
